# Patulin induced neuronal cell damage in human neuroblastoma SH-SY5Y cells

**DOI:** 10.1016/j.toxrep.2024.101886

**Published:** 2025-01-23

**Authors:** G.V. Jayashree, P. Rachitha, Vinay B. Raghavendra, Hemanth Kumar Kandikattu

**Affiliations:** a Biochemistry and Nanoscience department, Defense Food Research Laboratory, Mysore 570011, India; b Department of Biotechnology, Teresian College, Mysore 570011, India

**Keywords:** Patulin, Mycotoxins, SH-SY5Y, Mitochondrial damage, DNA damage

## Abstract

Patulin, a mycotoxin produced by fungal species, is found in fruits and their derivatives. Exposure to it can lead to cognitive deficits and neurodegenerative disorders. Understanding its mechanisms is crucial for assessing risks in food, emphasizing the need for strict food safety regulations to protect public health. In this study SH-SY5Y, a human neuroblastoma cell line was challenged with the mycotoxin patulin. Patulin was treated to the cells for 24 h at 25–2000 nM, concentrations respectively. The results obtained demonstrate the cytotoxicity as assessed by the MTT and LDH leakage assays with an IC50 at a dose of 500 nM. The light microscope images showed a decreased in neurites size with increase in doses of patulin. The patulin treatment showed a decrease in antioxidant enzymes SOD and catalase levels and an increase in ROS and lipid peroxidation levels. Patulin treatment also showed a decrease in mitochondrial membrane potential and mitochondrial damage, with vacuolation of mitochondria visualized by transmission electron microscope. Patulin treatment also showed DNA damage observed by comet assay. The study demonstrates that patulin induces cellular damage, and induces oxidative stress, apoptosis, mitochondrial and DNA damage.

## Highlights

• Patulin neuro toxicity studies in SHSY5Y cells.
• Patulin exposure shows genotoxicity observed by comet assay.
• Patulin damages Mitochondrial cells confirmed by TEM images.
• Patulin decreased the antioxidant enzymes SOD and catalase levels and an increase in ROS and lipid peroxidation levels.

## Graphical Abstract

**Figure:**
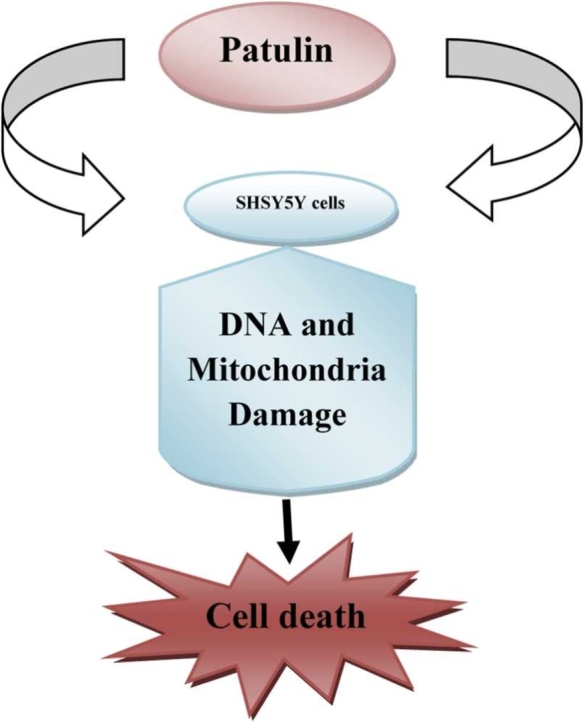

## 1 Introduction

Patulin is a toxin produced by *Penicillium*, *Aspergillus*, *Byssochlamys*, and *Alternaria*. It is one of the most common mycotoxins detected in fruits and their products [1]. Patulin may cause acute toxicity, such as nausea, vomiting, and diarrhoea, as well as long-term health consequences, such as immunotoxicity and genotoxicity. Patulin has been found in several dehydrated goods, such as tomatoes and other fruit crops. Notardonato et al. [2], [3]. Patulin, a natural antibacterial agent, was initially used to treat nose infections and common colds in the 1960’s due to its antiviral, antiprotozoal, and antibacterial properties [4]. However, in 1944, it was discovered to be hazardous in preclinical animal studies [5]. Patulin mycotoxin causes immediate symptoms, gastrointestinal distress, nausea, vomiting, and long-term damage to the kidneys, liver, and immune system [6]. Fruit and fruit juices are the most common commodities contaminated with patulin in Europe and North America. Apple and grape juices contain noticeable levels of patulin, which is a possible hazard to humans. There are now 11 nations with 30–50 ppb regulation restrictions on the amount of patulin acceptable in fruit juice [7].

Current studies have shown that patulin has negative health effects such as hepatotoxicity, gastrointestinal changes, and immunotoxicity [8]. When compared to the toxicity of other mycotoxins like ochratoxin A, patulin’s immunotoxicity can be considerably more detrimental [9]. Furthermore, patulin is a low molecular weight, extremely polar molecule, which presents numerous analytical problems for identification [10]. Patulin is categorized as non-carcinogenic but has been associated with adverse effects on the central nervous system [11]. Additionally, mycotoxins produced by fungal species are widespread and may pose greater health risks than insecticides. Exposure to these mycotoxins occurs through food, inhalation, or contact, leading to various health issues, including kidney damage, neurological disorders, and cancer [12].

Patulin, a mycotoxin, has been shown to have neurotoxic characteristics, raising concerns about its effects on the central nervous system. Patulin exposure causes a variety of negative neurological consequences, including oxidative stress, cellular damage, and poor neuronal health. Patulin may cause neurodegenerative reactions, such as apoptosis in brain cells and changes in protein expression [13]. Animal studies have also shown cognitive deficits and behavioural alterations in response to patulin exposure, indicating a possible role in neurodevelopmental and neuropsychiatric diseases [14]. Understanding patulin neurotoxicity is critical, considering its widespread presence in food and the potential long-term health consequences. Although previous studies have shed light on patulin's cytotoxic effects on various cell lines, there remains a significant knowledge gap regarding the specific pathways through which patulin induces toxicity in neuroblastoma cells via oxidative stress and apoptosis. Investigating the precise mechanisms by which patulin causes neurotoxicity, particularly in the SH-SY5Y neuroblastoma cell line, is essential for advancing our understanding of its effects.

The ultimate goal of this work is to fulfil the current research gap by examining the processes that underlie the neurotoxicity caused by patulin in SH-SY5Y cells. The aim of this study is to assess the function of oxidative stress and apoptosis in mediating the harmful effects of patulin, thereby advancing our knowledge of its influence on neuroblastoma and potentially informing future therapeutic approaches.

## 2 Materials and methods

### 2.1 Chemicals and reagents

Patulin was procured from Sigma, DMEM-F12 (Dulbecco's Modified Eagle Medium/Nutrient Mixture F-12) from HIMEDIA (Bangalore, India), penicillin and streptomycin solution, rhodamine 123, MTT (3-(4,5-dimethylthiazol-2-yl)-2,5-diphenyltetrazolium bromide.), 2',7'-DCFH_2_DA (2′,7′-Dichlorodihydrofluorescein diacetate.), RIPA buffer, protease, and phosphate inhibitor cocktail were obtained from Sigma (St. Louis, MO, USA) and all other chemicals were of analytical grade, procured from Rankem (Bangalore, India).

### 2.2 Cell culture and treatments

The SH-SY5Y human neuroblastoma cells were procured from the National Centre for Cell Sciences, Pune, India. The cells were equally seeded into flasks, Petri plates or dishes in 1:1, DMEM/F-12 mixture supplemented with 10 % FBS (Fetal Bovine Serum), 2m ML-glutamine, antibiotic and antimycotic solution (Sigma, St. Louis, MO, USA) in a humid atmosphere of 5 % CO_2_and 95 % air at 37 ºC. All the experiments were carried out in 0.5 % serum media. Cells were treated with different concentrations of patulin for 24 h.

### 2.3 Cell viability assay

The metabolic status of the mitochondria in SH-SY5Y cells was analysed by MTT [3-(4,5-dimethylthiazol-2-yl)-2,5-diphenyltetrazolium bromide] assay. The principle of the assay is based on the cleavage of tetrazolium salts by mitochondrial succinate reductase in viable cells to form formazan dye. The cells were cultured in 96-well plates at a density of 1 × 10 ^4^ cell/ml well and grown for 24 h and then treated with patulin (25, 50, 100, 250, 500, 750, 1000, 1250, 1500, 2000 nM). After 24-h incubation, MTT (0.5 mg/ml) was added to each well and incubated for 2 h at 37 ºC; 100 µl of DMSO was added to dissolve the formazan crystals [15]. The absorbance was then measured at 540 nm using a VERSA max Hidex plate chameleon™V (Finland) instrument and the cell viability was expressed as percent of control.

### 2.4 Lactate dehydrogenase (LDH) leakage assay

Cytotoxicity was quantified by measuring the extent of plasma membrane damage by means of an LDH leakage using enzyme detection kit (Agappe-11407002, Mysore, India) according to the manufacturers’ instructions. The LDH assay is based on the increase in the activity of the enzyme in the medium due to the leakage of cytosolic LDH following plasma membrane damage. The enzyme activity was measured through the oxidation of lactate to pyruvate with simultaneous reduction of nicotinamide adenine dinucleotide (NAD^+^) and optical density was recorded at a wavelength of 340 nm. The rate of increase in enzyme activity due to the formation of reduced nicotinamide adenine dinucleotide (NADH) is directly proportional to the LDH leaked in the medium. The SH-SY5Y cells were plated at a density of 5 × 10 ^4^ cells/well on 24-well plates and after 24 h of adherence the cells were subjected to the treatments with patulin (50, 100, 250, 500 nM) [16]. After the treatment period, 10 µl of cell lysis solution (2 % Triton X-100) was added to the untreated cells, which were selected as the total LDH activity. The cells were separated by centrifugation at 2500 rpm for 5 min at 4 ºC and the supernatant was analysed for LDH activity.

### 2.5 Morphological changes

The cells were seeded in Petri dishes (1 ×10^5^ cells/ml) followed by treatment with patulin for 24 h at different concentrations 50, 100, 250, 500 nM respectively. The cellular morphology was observed and photographed using a phase contrast microscope (Olympus, Japan) equipped with Cool SNAP® Pro colour digital camera.

### 2.6 Estimation of superoxide dismutase (SOD) and catalase (CAT)

SH-SY5Y cells (1 × 10^6^cells) were seeded in 75 cm^2^ flasks and treated as mentioned earlier. The cells were collected by trypsinization, washed twice with PBS, and the cell pellets were resuspended in ice-cold 50 mM potassium phosphate buffer, pH 7.4, containing 2 mM EDTA and 0.1 % Triton X-100. The cells were sonicated, followed by centrifugation at 13,000 × g for 10 min at 4 ^0^C to remove cell debris. The resulting supernatants were collected and the protein contents were measured by the Bradford method (1976) with BSA as standard. The activity of antioxidant enzymes such as superoxide dismutase (SOD) and catalase was estimated according to the kit supplier protocol (Randox, Cat no. SD. 125, Canada). Catalase (CAT) activity was estimated manually by measuring the decay of 6 mM H_2_O_2_ solution at 240 nm in a spectrophotometer [17].

### 2.7 Estimation of intracellular ROS

The cells were seeded in 24-well plates at a concentration of 4.0 × 10^4^cells/ml and treated as mentioned earlier. After treatments with 50, 100, 250, 500 nM Patulin, the oxidation-sensitive dye DCFH-DA (5 mg/ml) was added to the cells and incubated for 30 min [15]. The cells were then collected after washing twice with PBS and the intracellular ROS formation was detected fluorometrically with an excitation wavelength of 485 nm and an emission wavelength of 535 nm using Hidex plate chameleon™ V (Finland). For imaging, the cells were grown on cover slips which were pre-coated with poly L-lysine. After experimental treatments, the cells were treated with DCFH-DA as mentioned above and excess dye was removed by washing twice with PBS. The cells were imaged using fluorescence microscope (‘‘Olympus, Japan’’ equipped with Cool SNAP® Pro colour digital camera).

### 2.8 Estimation of lipid peroxidation

The lipid peroxidation was estimated by measuring malondialdehyde by the method described by Ohkawa et al. [18] with slight modifications. The SH-SY5Y cells were seeded in 75 cm^2^ flask at a concentration of 1.0× 101 × 10^6^ cells/ml and incubated at 37˚C. After reaching confluency, the cells were treated with 50, 100, 250, 500 nM patulin. The cells were harvested, washed with PBS, and sonicated in ice-cold 1.15 % KCl with 1 % Triton X-100. Then, 100 µl of the cell lysates were mixed with 0.2 ml of 8.1 % SDS, 1.5 ml of 20 % acetic acid (pH 3.5), and 1.5 ml of 0.8 % thiobarbituric acid. The volume was made up to 4.0 ml using distilled water and boiled for 90 min. After cooling, the contents were centrifuged at 1500 rpm for 10 min, the supernatants were separated, and the absorbance was measured at 532 nm.

### 2.9 Measurement of mitochondrial membrane potential (MMP)

The toxic effect of patulin on mitochondrial damage was determined by measuring the MMP using the fluorescent dye rhodamine 123 [15]. The cells were cultured in 24 well plates for the assay. After 50, 100, 250, 500 nM Patulin treatments, rhodamine 123 (10 µg/ml) was added to the cells and incubated for 1 h at 37 ^0^C. After washing twice with PBS, the cells were collected, and the fluorescence was detected at an excitation wavelength of 485 nm and an emission wavelength of 535 nm using Hidex plate chameleon ™ V (Finland).

### 2.10 Mitochondrial morphology by transmission electron microscope (TEM)

The cells were cultured in 75 cm^2^ flasks till the confluency reached and treated as mentioned earlier with 50, 100, 250, 500 nM patulin respectively, the cells were trypsinized, washed with cold PBS and cell pellets were fixed in 2.5 % glutaraldehyde (prepared in 0.1 mol/l PBS; pH 7.4) for 24 h at 4 ºC. The samples were post-fixed in 1 % OsO_4_ in PBS for 90 min at room temperature. Fixed samples were washed in several changes of PBS, dehydrated in graded alcohols and cleared by treating with propylene oxide for 15 min. The cells were left in 1:1 mixture of propylene oxide and embedding medium (TAAB Araldite, England) for overnight on a rotator [15]. The cells were then infiltrated by freshly prepared Araldite (Frasca and Parks1965; Johannessen1973) and transferred to flat silicon embedding moulds and kept for polymerization at 60 ºC for 48 h. Polymerized blocks were sectioned by ultramicrotome (Leica, Germany) and semi-thin Section (1 mm thick) were heat stained with toluidine blue and scanned under light microscope (Olympus, Japan). Then ultra-thin sections were made using ultramicrotome and mounted on formvar-coated nickel grids and stained with uranyl acetate followed by lead citrate. The grids were observed under a TEM (FEI TECANI G2 Spirit BioTWIN, Netherland).

### 2.11 Single cell gel electrophoresis (SCGE) assay

The toxic effect of patulin on DNA damage was assessed by alkaline comet assay. The cells (1 × 10^6^ cells) were seeded in 75 cm^2^ flasks and treated as described previously. The cells were collected, and an equal volume of cell suspension (4 × 10^5^) was mixed with 0.7 % (w/v) low melting agarose (LMA). The mixture was pipetted on to the frosted slides pre-coated with 1.0 % (w/v) normal melting agarose. After solidification of agarose, the slides were covered with another 100 ml of 0.7 % (w/v) LMA and immersed in lysis buffer (2.5 M NaCl, 100 mM EDTA, 10 mM Tris–HCl buffer, 0.1 % SDS and 1 % Triton X-100 and 10 % DMSO; pH 10.0) for 90 min to lyse the cellular and nuclear membranes. The slides were transferred onto an electrophoresis tank containing unwinding buffer (3 M NaOH, 10 mM EDTA; pH 13.0) for denaturing the DNA followed by electrophoresis for 20 min with an electric current of 25 V/300 mA. The slides were washed twice with neutralizing buffer (0.4 M Tris– HCl; pH 7.5) for 10 min and treated with ethanol for another 5 min. The slides were stained with 40 µl of ethidium bromide (20 µg/ml) and DNA damage was visualized by using fluorescence microscope (‘‘Olympus, Japan’’ equipped with Cool SNAP® Pro colour digital camera). The damage appeared as a ‘comet’ with fragmented DNA (tail) being separated from undamaged nuclear DNA (head) and measurements were made by RS Image® software to determine the tail length (µm). The results were expressed as percent inhibition of tail length.

### 2.12 Statistical analysis

The results were analysed by one-way ANOVA followed by a Tukey’s HSD-*post hoc* test. Significance level was set at a P-value < 0.05 and all comparisons were made against the control group.

## 3 Results and discussion

### 3.1 Patulin induced cytotoxicity

**Fig. 1:**
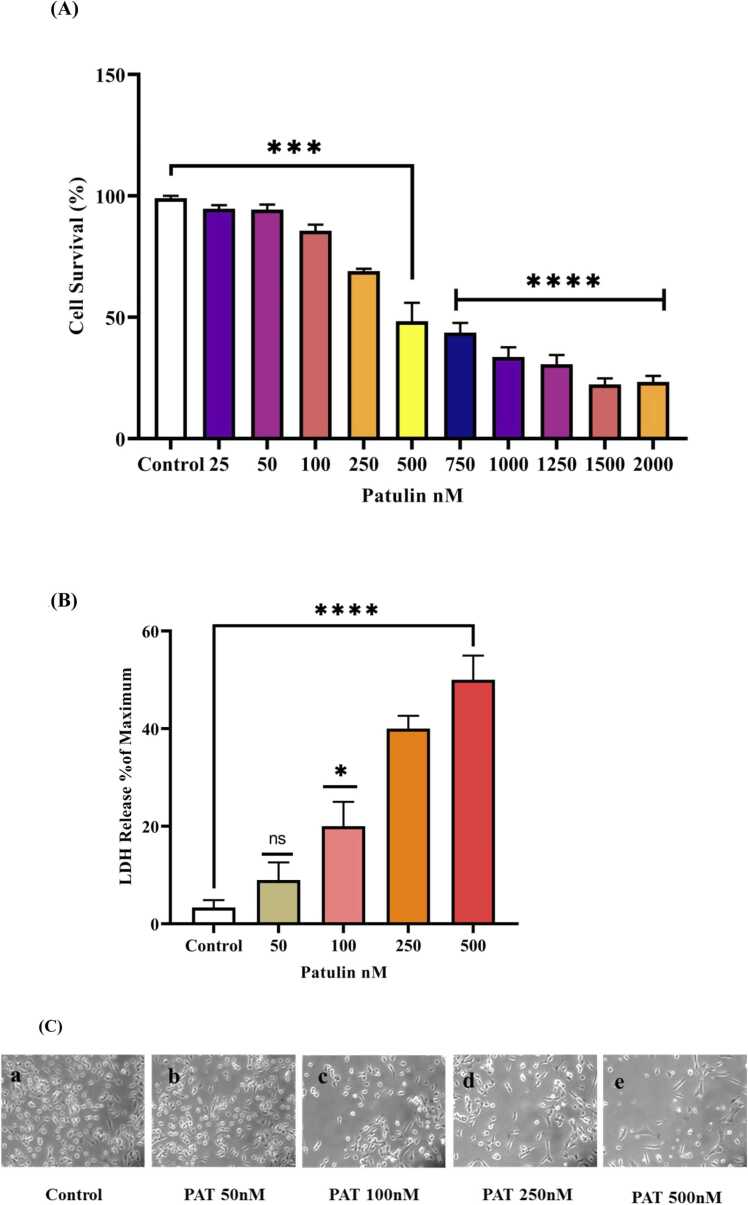
(**A)** Cytotoxic effects of patulin on SH-SY5Y neuronal cells, the cell viability was determined by MTT assay. Fig. 1**(B)** Cytotoxic effect of patulin on SH-SY5Y neuronal cell analysed by (LDH) lactate dehydrogenase leakage assay P < 0.05 versus control cells. Fig. 1**(C)** Effect of patulin induced morphological alteration in SH-SY5Y neuronal cells by phase-contrast microscopy. The data are represented as mean ± SD of three independent experiments. P < 0.05 versus control groups, *P < 0.05 versus control cells and different concentration 50, 100, 250, 500 nM of patulin treated group.

SH-SY5Y cells were exposed to different concentrations of patulin (25, 50, 100, 250, 500, 750, 1000,1250, 1500, 2000 nM) for 24 h and the MTT assay was carried out to determine the cell viability. The free radical-induced neuronal cytotoxicity was evaluated after patulin treatment at different concentrations. Patulin treatment decreased the cell proliferation in a dose-dependent manner with 50 % viability at 500 nM patulin challenge. This concentration was used for further assays (Fig. 1A). The MTT assay measures the metabolic activity of live cells and offers vital information about cell viability [19]. In SH-SY5Y cells treated with patulin, it is appropriate to expect a fall in MTT reduction, which will lead to a significant decrease in viable cell counts. The concept that patulin induces cytotoxicity by interfering with cellular metabolism and function is supported by this reduction, which is congruent with the morphological changes seen under bright field microscopy. The relationship between the quantity of patulin given and the pace at which cell viability decreases would be highlighted by the concentration-dependent effects of the MTT experiment.

**Fig. 2:**
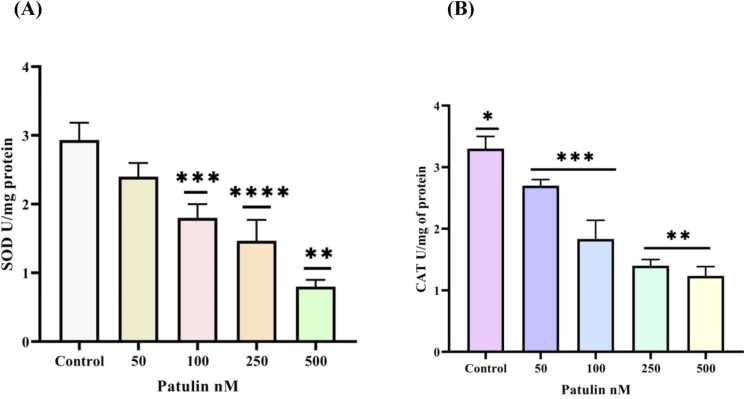
(**A) (B)** Effect of SOD and catalase enzyme activities in SH-SY5Y cells challenged with different concentration 50, 100, 250, 500 nM of patulin treated group. The data are represented as mean ± SD of three independent experiments. P < 0.05 versus control groups, *P < 0.05 versus control cells and different concentration 50, 100, 250, 500nM of patulin treated group.

In order to investigate the patulin induced cell damage LDH leakage was assessed. The lactate dehydrogenase (LDH) release assay serves as an important indicator of cellular membrane integrity and cytotoxicity [20]. LDH leakage was dose-dependently increased with patulin challenge of SH-SY5Y human neuroblastoma cells. The results observed demonstrate 48 % LDH release of the total enzyme after exposure to 500 nM patulin treatment which indicates that patulin induces cytotoxicity in the SH-SY5Y cells as shown in Fig. 1B. Bright-field microscope images reveal the morphological changes induced by patulin. The images clearly show the cell shrinkage and disappearance of the cellular integrity Fig. 1C. According to the study, increased patulin levels have cytotoxic effects by compromising the integrity of cellular membranes. This implies that patulin exposure may cause apoptotic signaling pathways to be triggered and cellular homeostasis to be upset. The relationship between patulin content and LDH leakage emphasizes the necessity of closely monitoring mycotoxin levels in food products in order to reduce the risk of neurotoxicity.

### 3.2 Effect of patulin on antioxidant enzymes

The antioxidant enzymes superoxide dismutase (SOD) and catalase (CAT), which are essential for the detoxification of free radicals produced by oxidative stress in cells, were assessed in the current study. The findings demonstrated that SOD activity was 0.8 % lower than that of the control group, which showed 2.8 % activity. Similarly, CAT activity dropped by 1.2 % as opposed to 3.7 % for the control. This indicates that the influence of patulin treatment has resulted in a notable deterioration of the antioxidant defence mechanisms. It has been discovered that the mycotoxin produced by molds, decreases the activity of superoxide dismutase (SOD) and catalase (CAT) enzymes, which negatively impacts cellular stress responses. These enzymes are essential for detoxifying hydrogen peroxide into water and oxygen and for changing superoxide radicals into hydrogen peroxide. Our findings are also supported by the research done by Zhang et al. [21] and Han et al. [22]. A variety of illnesses, including cancer, cardiovascular disease, and neurological diseases, may result from the disruption of these enzymes, which could also cause cellular damage and dysfunction [23], [8], [24]. It is essential to comprehend the mechanisms underlying these consequences in order to create mitigation or preventative plans. Additional investigation may be necessary to examine the enduring effects of decreased SOD and CAT activity.

### 3.3 Patulin-induced ROS generation and lipid peroxidation

**Fig. 3:**
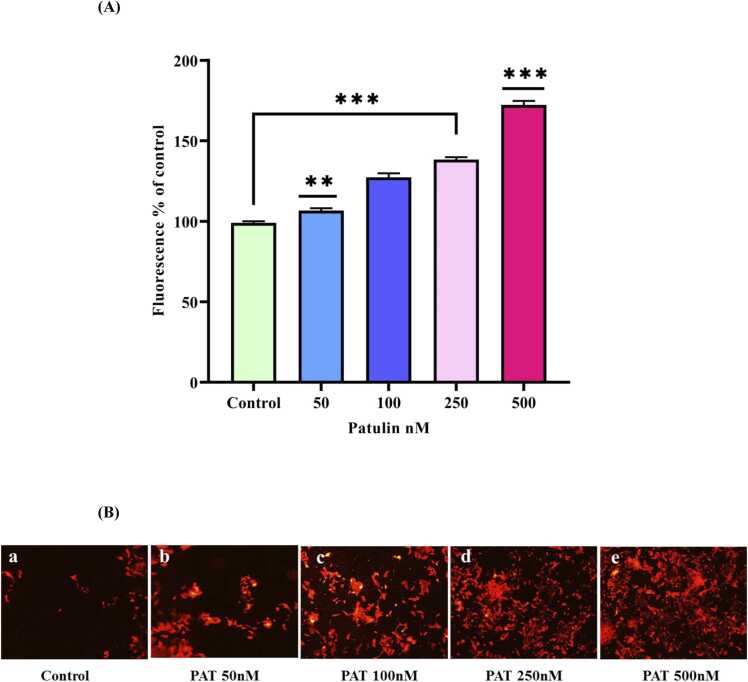
(**A)** Estimation of intracellular ROS production using 2',7'-DCFH_2_DA using spectrofluorometer. Fig. 3**(B)** The ROS production in SH-SY5Y cells was monitored by a fluorescent microscope (Olympus, Japan) (a) Control cells without any treatment, (b) 50 nM patulin treatment, (c) 100 nM patulin treatment, (d) 250 nM patulin treatment, (e) 500 nM patulin treatment.

This work used the fluorescent probe DCFH-DA to determine how patulin affects the production of reactive oxygen species (ROS) in human neuronal cells. It was discovered that when cells treated with 500 nM of patulin the fluorescence intensity, which is indicative of ROS levels, increased drastically by 169 % compared to a control group, which displayed a ROS fluorescence intensity of 99 %. The strong oxidizing properties of patulin, which have been previously described in a variety of cell types, including human hepatocellular carcinoma and Chinese hamster ovary cell lines, are highlighted by this substantial increase in ROS generation [25], [21]. This study found that after 500 nM of patulin administration, there was a significant rise not just in ROS generation but also in lipid peroxidation products Figs. 3A and 3B.

### 3.4 Effect of patulin on MMP

**Fig. 4:**
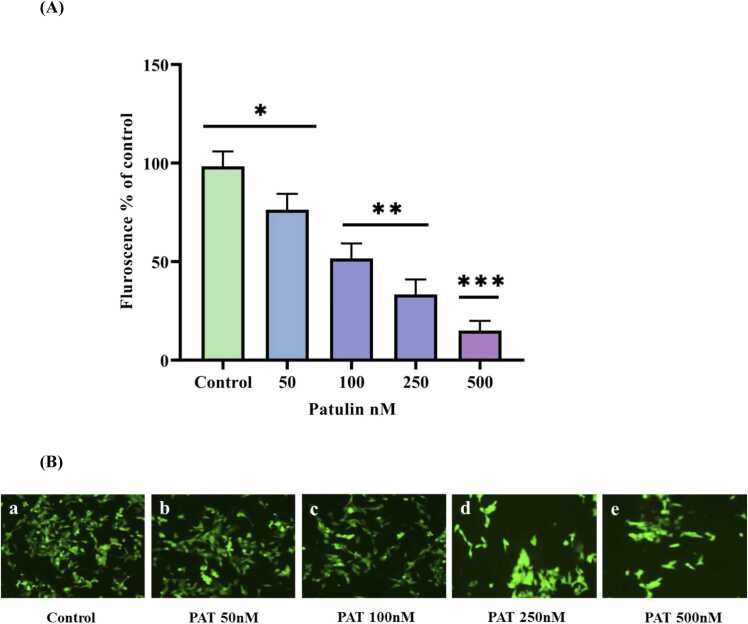
(**A)** Estimation of mitochondrial membrane potential in SH-SY5Y cells treated with patulin. The fluorescence intensity was determined using spectrofluorometer. Fig. 4**(B)** The membrane potential was monitored by a fluorescent microscope (Olympus, Japan) (a) Control cells without any treatment, (b) 50 nM patulin treatment, (c) 100 nM patulin treatment, (d) 250 nM patulin treatment, (e) 500 nM patulin treatment. The data are represented as mean ± SD of three independent experiments. P < 0.05 versus control groups, *P < 0.05 versus control cells and different concentration 50, 100, 250, 500 nM of patulin treated group.

Rhodamine 123 was used to measure the extent of mitochondrial damage in SH-SY5Y human neuroblastoma cells. The results showed a considerable reduction in mitochondrial membrane potential (MMP), with a 20 % reduction detected after 500 nM of patulin treatment Figs. 4A and 4B. The ability of patulin to cause mitochondrial depolarisation, a critical marker of mitochondrial malfunction and damage, is shown by this drop in fluorescence intensity. These results were further supported by electron microscope analysis, which showed changes in mitochondrial morphology consistent with the biochemical outcomes. This suggests that structural defects are probably responsible for the cells' heightened oxidative stress and reduced ability to produce energy [26]. Such mitochondrial malfunction is important because it not only prevents the synthesis of ATP but also sets off a series of events that ultimately result in cell death, explaining the toxic effects of patulin on brain cells [27]. This knowledge highlights the significance of mitochondrial integrity for the health of neurons and implies that treatments aimed at protecting the mitochondria could lessen the negative consequences of exposure to patulin.

### 3.5 Effect of patulin on mitochondria structure and lipid peroxidation

**Fig. 5:**
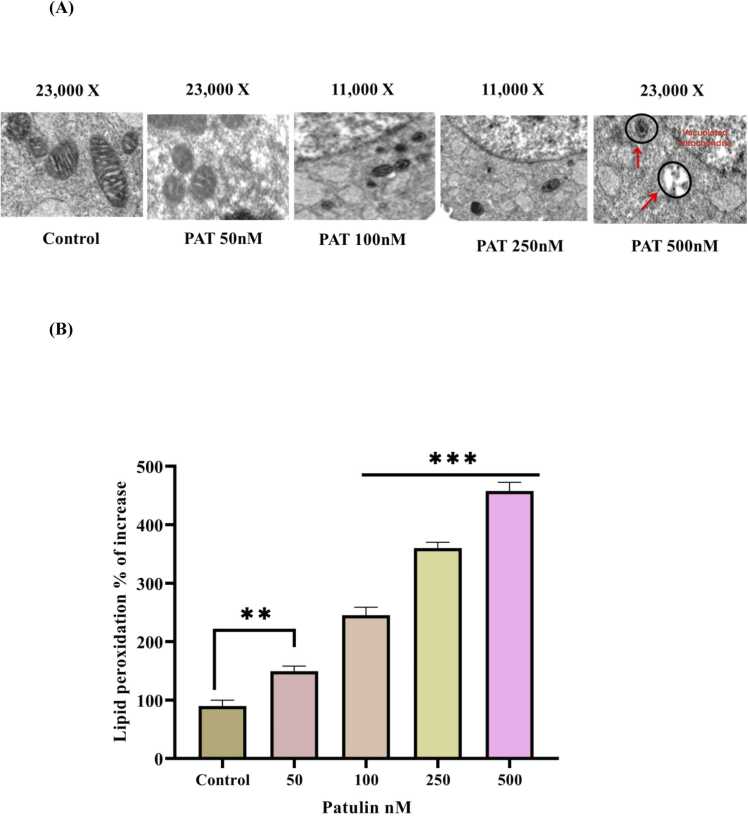
(**A)** Transmission electron-microscopic analysis of the mitochondrial integrity of SH-SY5Y cells. Fig. 5**(B)** Estimation of lipid peroxidation products by TBARS assay in SH-SY5Y cells treated with patulin. The data are represented as mean ± SD of three independent experiments. P < 0.05 versus control groups, *P < 0.05 versus control cells and different concentration 50, 100, 250, 500 nMof patulin treated group.

Transmission electron microscopy (TEM) of control cells showed mitochondria with regular shape and structure with proper cristae and well-defined membranes Fig. 5(A) 23000x magnifications. Patulin treatment dramatically altered the structure of mitochondria, and more than 85 % of the mitochondria were observed vacuolated and swollen and showed destruction in cristae. The damage was more pronounced at a 500 nM level of patulin compared to lower concentrations. The results of the transmission electron microscopy (TEM) analysis shed important light on the mitochondrial damage that patulin causes to SH-SY5Y human neuroblastoma cells. Notably, altered cristae and damaged membrane integrity—observed changes in mitochondrial morphology—strongly suggest impaired mitochondrial function. These findings are similar to the study conducted by the [28], ROS-mediated vacuolation visualized by TEM. Further supporting earlier results of a 20 % drop in mitochondrial membrane potential (MMP) with exposure to patulin, these structural alterations are frequently linked to mitochondrial depolarisation and death. Because it upsets the electrochemical gradient required for ATP synthesis, this decline is critical because it can seriously harm cellular energy metabolism and survival [29]. Patulin's cytotoxic actions involve more than just direct damage; they additionally trigger lipid peroxidation activities. Secondary products from lipid peroxidation, such as malondialdehyde (MDA), harm mitochondrial membranes and exacerbate their impairment of function and integrity [30]. Lipid peroxidation products are a well-known indicator of oxidative stress, suggesting that patulin not only causes the production of ROS but also causes subsequent cellular damage, such as the destruction of lipid membranes [31]. This is consistent with other research showing that patulin causes oxidative stress via processes including the production of reactive oxygen species (ROS) as well as the deficiency of antioxidant defence inside cells [32]. An increase in ROS may have harmful consequences, such as oxidative damage to lipids, proteins, and nucleic acids, which may impair cellular processes and exacerbate neurotoxicity [33]. The increased oxidative stress brought on by patulin may be a factor in the reduced viability and functionality of neurons Fig. 5(B). The sequence of events indicates that the underlying lipid peroxidation processes brought on by patulin exposure are the cause of the mitochondrial damage shown by TEM. The accumulation of lipid peroxidation products reduces mitochondrial metabolic function and increases oxidative stress-mediated SH-SY5Y neuronal cell injury.

### 3.6 Effect of patulin on DNA damage

**Fig. 6:**
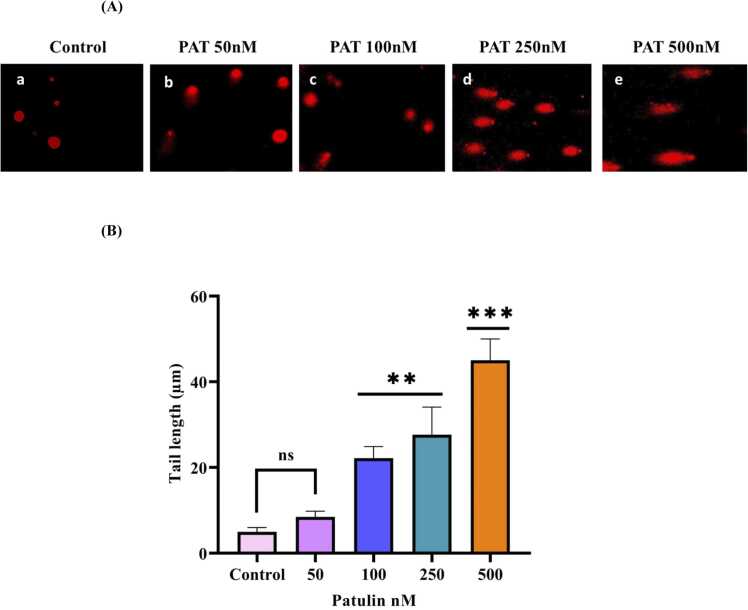
(**A)** and **(B)** Toxic effect a tail length of patulin on DNA damage induced by different concentration 50, 100, 250, 500 nM of patulin treated group in SH-SY5Y cells. (a) Control cells without any treatment, (b) 50 nM patulin treatment, (c) 100 nM patulin treatment, (d) 250 nM patulin treatment, (e) 500 nM patulin treatment. The data are represented as mean ± SD of three independent experiments. P < 0.05 versus control groups, *P < 0.05 versus control cells and different concentration 50, 100, 250, 500 nM of patulin treated group.

Comet assay was performed to assess the DNA damage induced by patulin in SH-SY5Y neuronal cells. The tail length of the comet in control was compared with that of treated samples. The results are presented in Figs. 6A and 6B. The tail length increased with increasing concentrations of patulin. The increased comet tail length, which indicates severe DNA damage, is consistent with previous results that mycotoxins, such as patulin, can disturb genomic integrity and increase the likelihood of oxidative stress-induced cellular malfunction and apoptosis [34]. Such DNA damage in brain cells may compromise vital cellular functions, resulting in neurotoxicity, which over time may show up as cognitive deficiencies or neurodegenerative disorders [35]. The noted dose-dependent association highlights the necessity of more research into the patulin concentration thresholds that cause DNA damage and the processes by which this mycotoxin interacts with cellular structures to cause genotoxicity.

## 4 Conclusion

The study reveals the neurotoxic effects of patulin, particularly on neuroblastoma cells. Patulin reduces cellular performance, disrupts energy metabolism, and increases susceptibility to oxidative stress, leading to DNA damage and cell death. The study also highlights the potential long-term neurotoxic effects of regular consumption of molds contaminated foods particularly patulin, which could contribute to neurodegenerative processes and cognitive deficits. The exposure levels used in the experiments reflect concentrations found in contaminated foods, raising concerns about the potential for long-term neurotoxic effects in consumers. The research emphasizes the need for strict monitoring of patulin levels in food to protect public health and the development of effective strategies for mitigating exposure to mycotoxins. The study calls for increased understanding of patulin's neurotoxic effects and risks associated with its occurrence in food sources to prevent adverse health outcomes and promote consumer safety.

## CRediT authorship contribution statement

**Raghavendra Vinay B:** Writing – review & editing, Visualization. **P Rachitha:** Writing – review & editing, Methodology. **Kandikattu Hemanth:** Writing – review & editing, Supervision, Methodology, Conceptualization. **GV Jayashree:** Writing – original draft, Conceptualization.

## Declaration of Competing Interest

The authors declare that they have no known competing financial interests or personal relationships that could have appeared to influence the work reported in this paper.

## Data Availability

Data will be made available on request.
